# Hot Tea Consumption and Esophageal Cancer Risk: A Meta-Analysis of Observational Studies

**DOI:** 10.3389/fnut.2022.831567

**Published:** 2022-04-11

**Authors:** Hui Luo, Hong Ge

**Affiliations:** Department of Radiation Oncology, The Affiliated Cancer Hospital of Zhengzhou University, Henan, China

**Keywords:** hot tea, esophageal cancer, meta-analysis, case-control study, risk

## Abstract

**Objective:**

Many laboratory studies have shown that tea consumption protected against the development of esophageal cancer (EC). However, in epidemiological studies, inconsistent or even contradictory results were frequently observed, especially when drinking tea at higher temperatures.

**Methods:**

We conducted a meta-analysis based on published observational studies to explore whether hot tea consumption was a risk factor of EC. Relevant studies were searched in PubMed, Embase, and Web of science up to October 13, 2021, and we also manually retrieved the literature in the included studies and recent reviews.

**Results:**

A total of 23 eligible reports were identified, including 5,050 cases and 10,609 controls, and a meta-analysis with Comprehensive Meta-Analysis (CMA) software (version 2.0) was conducted. A statistically significant increased EC risk was observed when drinking tea at higher temperature (odds ratios (ORs) = 1.79, 95% CI: 1.48–2.15, *p* = 0.00). Except for esophageal adenocarcinoma (EAC), this increased risk was also found in the majority of subgroups, which are the European and Australian populations.

**Conclusions:**

This meta-analysis showed that people who drank hot tea had a significantly increased risk of Esophageal squamous cell carcinoma (ESCC), but no significant association for EAC.

## Introduction

Esophageal cancer (EC) is the seventh most commonly diagnosed cancer and the sixth cause of cancer mortality worldwide, with approximately 70% of cases occurring in men and a 2- to 3-fold difference in the incidence and mortality rates between different regions (1). Esophageal squamous cell carcinoma (ESCC) and esophageal adenocarcinoma (EAC) are the two main histologic subtypes, which have been reported to be associated with different risk factors (2). The ESCC is the predominant histological type worldwide, accounting for over 80% of all esophageal cancers (3). However, currently, the EAC subtype is starting to be the dominant one in Australia, the United States, and some Western European countries, moving the ESCC subtype to second in incidence in these regions (4). Heavy alcohol drinking or smoking and their synergistic effects are the major established risk factors for ESCC in Western countries (1). However, in lower-income countries such as those in the “Asian EC Belt”, which mainly refers to Kazakhstan, Iran, Turkey, and northern and central China, the major risk factors have yet to be elucidated. These are high-risk areas with EC incidence rates reaching even more than 100 per 100,000 population (4). The prognosis of EC is poor, with the average 5-year survival rates between just 15 to 25% after comprehensive treatment, resulting in 509,000 deaths from EC in 2018 (5, 6). Prevention of cancer at the early stage, therefore, plays a key role in reducing the global burden of EC.

Although the definitive mechanism of EC development is still unclear, many studies have shown that dietary habits are significantly associated with the development of EC (7–9). For example, drinking tea has been reported to inhibit the occurrence of esophageal tumors (10, 11). Tea, a popular beverage worldwide, which is made using the dried leaves of the plant Camellia sinensis, is mainly consumed in the form of black and green tea. Green tea is rich in polyphenols, which have been extensively studied as a cancer chemo-preventive agent. Epigallocatechin gallate (EGCG), the most abundant and active compound in tea, was reported to block cancer progression (12, 13). In epidemiological studies, the association between tea drinking and reduced EC risk was also confirmed by several case-control studies (11, 14), prospective cohort studies (15), and meta-analyses (16–18). However, opposite or non-significant conclusions were also reported, especially when drinking tea at higher temperatures (19–22).

Tea beverages are usually a mixture of boiling water and tea leaves. So, the potential thermal injury should be considered because very hot beverages are identified by the International Agency for Research on Cancer (IARC) monograph as Category 2A carcinogens for ESCC (23). Given the inconsistent relationship and that individual studies may be underpowered to accurately detect the potential risk of EC and hot tea consumption, we performed a meta-analysis to evaluate this correlation more accurately.

## Materials and Methods

### Literature Search Strategy

Published studies related to hot tea consumption and EC risk were searched in the databases of Embase, PubMed, and Web of Science up to October 13, 2021. The search terms used were as follows: (a) “hot” or “high temperature,” (b) “esophageal cancer” or “esophageal carcinoma” or “esophageal neoplasm,” (c) “tea” or “beverage” or “drinking;” these searched keywords were combined with “and/or” without restrictions. In addition, we also retrieved papers in the reference lists and recent reviews.

### Selection Criteria of the Study

The studies included in the meta-analysis should satisfy all of the following criteria: (a) It was a case-control or cohort study; (b) The ORs or adjusted OR values and relevant corresponding 95% CIs were reported, or provided sufficient information to calculate it; (c) The study was about tea individually or tea was definitely included in the study; (d) The study tested the relationship between EC risk and tea temperature; (e) The diagnoses were confirmed as EC; and (f) It was published in English.

### Assessment of Study Quality

The quality of included studies was evaluated by two reviewers independently with the Newcastle-Ottawa Scale (NOS) recommended by the Cochrane Non-Randomized Studies Methods Working Group (24). This was a nine-star scale that allocated stars based on the selection process (0-4 stars), the comparability (0–2 stars), and the outcomes assessment of study participants (0-3 stars). Studies with 0–3, 4–6, and 7–9 stars were defined as low, moderate, and high-quality studies, respectively.

### Data Extraction

Two researchers completed the selection process independently to review the eligibility of all the studies and extract the required information, including the first author's family name, year of publication, country, study design, type of cancer, gender of subjects, number of cases, and controls, temperatures of tea, ORs and 95% CIs, and adjusted confounders. Reduplicative references were deleted using the Endnote software, and then, we read the titles and abstracts to check whether the articles met the inclusion criteria. If it is difficult to determine the eligibility of one paper according to the title and abstract solely, the full text is downloaded and checked for the final decision. Any disagreements about research choices among researchers were resolved through discussion.

Adjusted ORs were extracted preferentially to non-adjusted ones; however, unadjusted ORs and 95% CIs were accepted when adjusted ORs were not provided. The one with the most adjusted variables was selected when more than one adjusted OR was reported. Where multiple risk estimates were available in the same study, for example, studies providing ORs for both ESCC and EAC, they were included as two separate studies. Where different temperatures were reported, the highest qualitatively described temperature was chosen, such as “hot,” “scalding,” and “very hot”.

### Statistical Analysis

The analyses were performed by the software of Comprehensive Meta-Analysis (CMA) version 2.0, like combing the effect size of ORs and 95% CIs, generating forest plots and funnel plots, and determining whether there was a statistical association. The statistical significance threshold was set at *p* < 0.05. The heterogeneity was assessed with chi-square based on Cochran's Q statistic (25), and the I^2^ statistic, I^2^ = 0–25%, 25–50%, 50–75%, and 75–100% were considered as no, moderate, large and extreme heterogeneity (26). Generally, the fixed-effects model was selected for analysis, however, when an I^2^ > 50% existed, the random-effects model was used to estimate OR and CI. Subgroup analysis was performed according to cancer types, country, gender, study design, and confounders to identify the cause of heterogeneity and minimize it. Besides, Sensitivity analysis by omitting one study in turn with CMA was conducted to test the robustness of the main results. Specifically, if there was no significant change in the results after the exclusion, it indicated low sensitivity and reliable results. On the contrary, if there was a significant difference or even opposite conclusion after the exclusion, it implied high sensitivity. Both Egger's weighted linear regression test and Begg's rank correlation test have been proposed for publication bias in the funnel plot and asymmetry of the funnel plots indicated potential publication bias (27, 28).

## Results

### Literature Search

**Figure 1:**
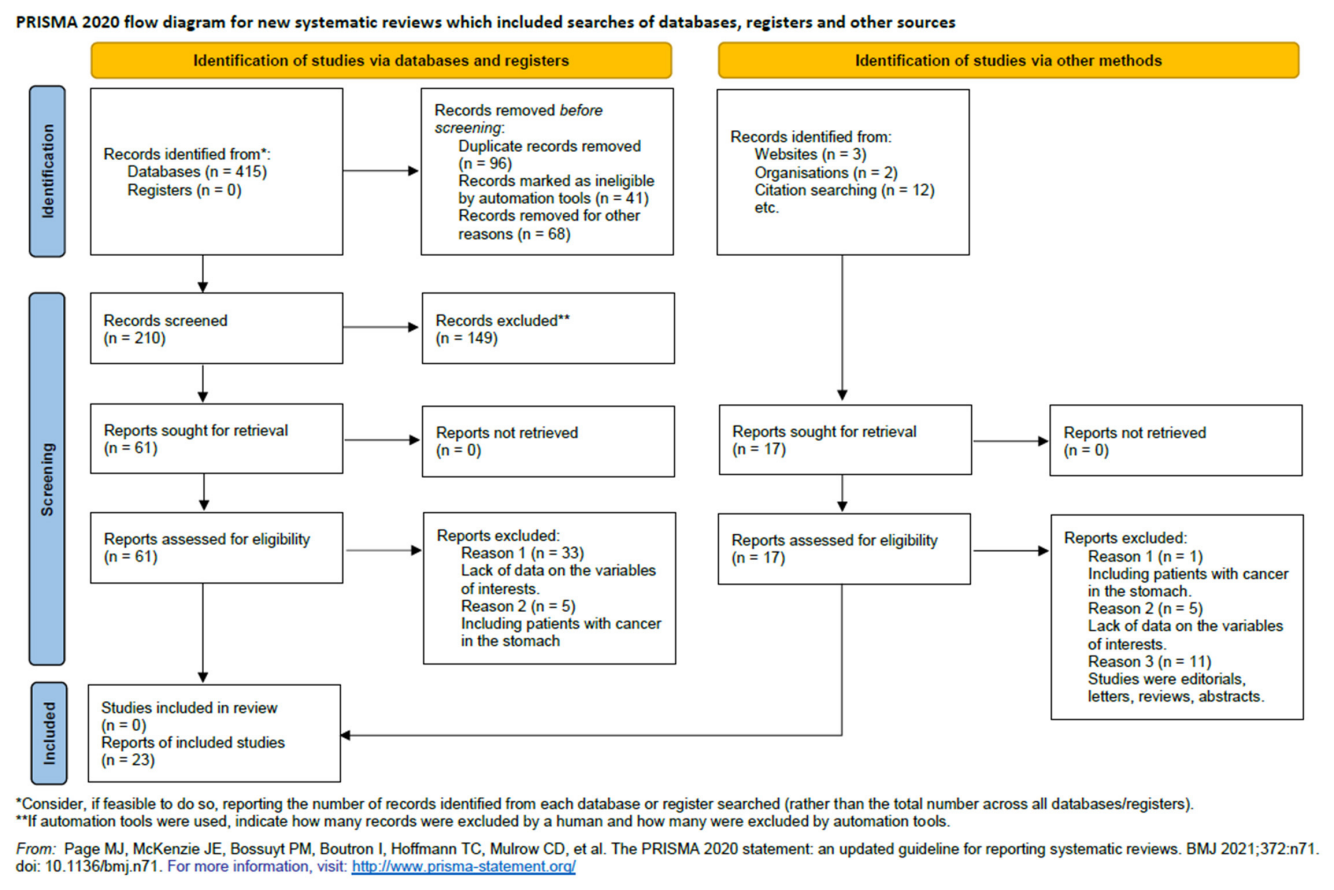
Flowchart of the studies selection process.

We retrieved 210 unique articles from the databases of PubMed, Embase, and Web of Science, 61 of which were identified as potentially relevant. After reviewing the full text, we determined that 23 reports met our inclusion criteria (14, 19, 20, 29–48). The literature search and study screening process are shown in Figure 1.

### Characteristics of Included Studies

Finally, 23 articles (34 individual studies) were identified in this meta-analysis including 5,050 cases and 10,609 controls. The detailed characteristics of the included studies are shown in Table 1. All the 23 articles included were published in English, and the cases were histologically, pathologically, or cytologically confirmed as EC. In these studies, 20 were population-based case-control studies (PB-CC), eight were hospital-based case-control studies (HB-CC), and 6 were prospective cohort studies (PS-CH). There were 22 studies performed in Asia, four in the Americas, four in Europe, three in Australia, and one in Africa. Twenty-five of the studies reported adjusted ORs, 95% Cis, and the adjusted confounders. Twenty-five of the studies controlled smoking or alcohol in models, and 22 controlled age in models. Twenty-two studies reported results for males and females together, 6 reported the results for women separately, and 6 reported results for men only.

**Table 1:** Characteristics of studies included in the meta-analysis.

**Reference**	**Location**	**Study type subjects/ participants**	**Cancer type**	**Gender**	**Exposure**	**OR (95%CI)**	**Effect estimates**	**NOS**
Cook–Mozaffari et al. (29)	Iran	PS–CH 344/688	EC	M F	Never Hot Never Hot	1.0 1.59 (1.14–2.27) 1.0 1.89 (1.22–2.94)	NR	8
Gao et al. (14)	China	PB–CC 217/920	EC	M F	Non–tea drinker Burning–hot Non–tea drinker Burning–hot	1.0 3.09 (1.94–4.93) 1.0 2.0 (0.75–5.07)	Age, education, birthplace, cigarette smoking, and alcohol intake (men only).	7
Srivastava et al. (30)	India	PB–CC 170/170	EC	M/F	Hot Very hot	1.0 1.74 (1.65–2.89)	NR	6
Kinjo et al. (31)	Japan	PS–CH 96/344	EC	M/F** M* F*	Not hot Hot Hot Hot	1.0 1.5(1.1–2.0) 1.5 (1.1–2.0) 1.8 (1.1–2.9)	* Age, prefecture, and occupation. **Age, sex, prefecture, occupation, vegetable intake and tobacco and alcohol use	9
Castellsagué et al. (32)	South America	HB–CC 47/77	ESCC	M/F M F	Cold/warm Hot Very hot Hot Very hot Hot Very hot	1.0 0.66 (0.35–1.25) 3.73 (1.41–9.89) 0.85 (0.37–1.95) 8.73 (1.95–39.10) 0.58 (0.16–2.07) 2.20 (0.42–11.56)	Age group, sex, hospital, residency, education and tobacco and alcohol use	8
Nayar et al. (34)	India	HB–CC 150/150	EC	M/F	warm Hot Burning hot	1.0 1.11 (0.62–1.96) 1.27 (0.60–2)	NR	7
Cheng et al. (33)	England	PB–CC 32/32	EAC	F	Warm Hot Very hot	1.0 0.75 (0.32–1.76) 0.51 (0.18–1.45)	NR	8
Terry et al. (36)	Sweden	PB–CC 356/815	167 ESCC 189 EAC	M/F	ESCC None, cold, lukewarm Hot Very hot EAC Hot Very hot	1.0 1.0 (0.6–1.6) 0.8 (0.4–1.8) 0.7 (0.5–1.1) 0.6 (0.3–1.3)	Age, sex, BMI, socioeconomic status, tobacco and alcohol use, gastroesophageal reflux symptoms, frequency of hot beverage drinking, energy and fruit and vegetable intake	8
Sharp et al. (35)	England	PB–CC 75/86	ESCC	F	Warm Hot Very hot	0.34 (0.13–0.88) 0.75 (0.38–1.47) 1.0	Slimming diet, breakfast, salad, smoking, regular use of aspirin, aspirin center and temperature of tea/coffee	7
Onuk et al. (37)	Turkey	HB–CC 44/100	EC	M/F	Not hot Hot	1.0 8.7 (2.5–30.2)	Tobacco use, fruit, vegetable, coffee, pickle intake and type of bread	8
Wu et al. (40)	China	PB–CC 1154/2884	EC	M/F	High–risk area: Never drinking Normal temperature High temperature Low–risk area: Never drinking Normal temperature High temperature	1.0 1.0 (0.7–1.3) 2.2 (1.6–5.3) 1.0 1.3 (0.9–1.7) 4.2 (2.3–7.6)	Age, gender, education level, family history of cancer, BMI, tobacco and alcohol use; green tea consumed was adjusted for tea temperature	8
Islami et al. (38)	Iran	PB–CC 300/571	ESCC	M/F	Warm or lukewarm Hot Very hot	1.0 2.07 (1.28–3.35) 8.16 (3.93–16.91)	Ethnicity, education, tobacco or opium use, alcohol use, vegetable intake, black tea consumption, green tea consumption and tea temperature	7
Joshi et al. (39)	India	HB–CC 44/66	EC	M/F	Warm Hot Very hot	1.0 0.26 (0.29–1.09) 0.27 (0.25–1.28)	NR	7
Ren et al. (42)	US	PS–CH 50/173	ESCC EAC	M/F	Never drinking Hot (ESCC) Hot (EAC)	1.0 0.57 (0.30–1.07) 0.97 (0.67–1.41)	Age, sex, tobacco smoking, alcohol drinking, BMI, education, ethnicity, fruit and vegetables, red meat, white meat, and calories	7
Ibiebele et al. (41)	Australia	PB–CC 123/196	ESCC EAC EGJAC	M/F	ESCC Room temperature to Luke–warm Warm Hot Very hot EAC Room temperature to Luke–warm Warm Hot Very hot EGJAC Room temperature to Luke–warm Warm Hot Very hot	1.0 1.72 (0.64–4.60) 0.70 (0.30–1.65) 1.28 (0.51–3.19) 1.0 1.56 (0.67–3.61) 0.75 (0.37–1.54) 0.51 (0.21–1.22) 1.0 0.92 (0.42–2.03) 0.70 (0.36–1.37) 0.61 (0.29–1.31)	Age, gender; smoking, alcohol intake; heartburn and acid reflux symptoms, BMI, educational, fruit and vegetable intake	8
Chen et al. (43)	China	HB–CC 93/144	ESCC	M/F	Never Warm Hot Very hot	1.0 0.76 (0.36–1.32) 2.41 (1.53–4.17) 3.83 (2.23–6.54)	NR	6
Tang et al. (44)	China	HB–CC 359/380	EC	M/F	Low or mild High	1.0 2.86 (1.73, 4.72)	Age, gender, education, BMI, smoking, alcohol drinking, family history of cancer, vegetables and fruit	8
Zhao et al. (45)	China	PB–CC 22/68	ESCC	M/F	Warm Hot	1.0 2.50 (0.93–6.75)	Age, smoking, alcohol drinking, family history of EC, fruit intake, education, and BMI	
Tai et al. (47)	China	PB–CC 167/167	ESCC	M/F	Low or mild (<60°C) High (≥60°C)	1.0 2.23 (1.45–2.90)	Age, sex, education, BMI, smoking status, alcohol drinking, family history of cancer, vegetables and fruits	8
Hamrah et al. (46)	Afghanistan	PB–CC 38/130	EC	M F	Cold/lukewarm Hot Hot	1.0 1.14 (0.55–2.37) 1.13 (0.54–2.35)	NR	5
Middleton et al. (48)	Kenya	HB–CC 178/142	ESCC	M/F	Warm Hot Very hot	1.0 1.40 (0.97–2.03) 3.66 (2.10–6.50)	Age, sex, study phase, interviewer, tobacco and alcohol consumption; family history of EC; education level	7
Yang et al. (19)	China	PB–CC 250/280	ESCC	M	Never Warm Hot Very hot	1.0 1.29 (0.99–1.69) 1.47 (1.14–1.91) 2.15 (1.52–3.05)	Age, marital status, education, occupation, family wealth score, BMI, sum of missing and filled teeth, number of teeth brushing per day, smoking pack–years, alcohol consumption, family history of EC	7
Yu et al. (20)	China	PB–CC 111/1231	EC	M F	Less Than Weekly Weekly Warm Hot Very hot Less Than Weekly Weekly Warm Hot Very hot	1.0 0.93 (0.70–1.24) 1.17 (0.91–1.50) 1.30 (1.05–1.59) 1.55 (1.19–2.02) 1.0 0.52 (0.21–1.27) 1.04 (0.54–2.02) 1.30 (0.74–2.29) 1.13 (0.51–2.51)	Age, education, marital status, household income, physical activity, intake of red meat, fruits and vegetables, BMI, family history of cancer, menopausal status (for women only), tobacco smoking, alcohol consumption	6

BMI, body mass index; EAC, esophageal adenocarcinoma; EC, esophageal cancer; EGJAC, esophageal-gastric junction adenocarcinoma; ESCC, esophageal squamous cell carcinoma; F, female; HB-CC, hospital-based case control studies; M, male; NOS, Newcastle-Ottawa Scale; NR, not reported; OR, odds ratio; PB-CC, population-based case control studies; PS-CH, prospective cohort studies. *Means the group of Male or Female, **means the group of Male and Female.

The qualities of the studies included were all evaluated by the NOS method, and all eligible studies scored highly (with five stars or more, Table 1).

### Overall Hot Tea Drinking and EC Risk Analysis

**Figure 2:**
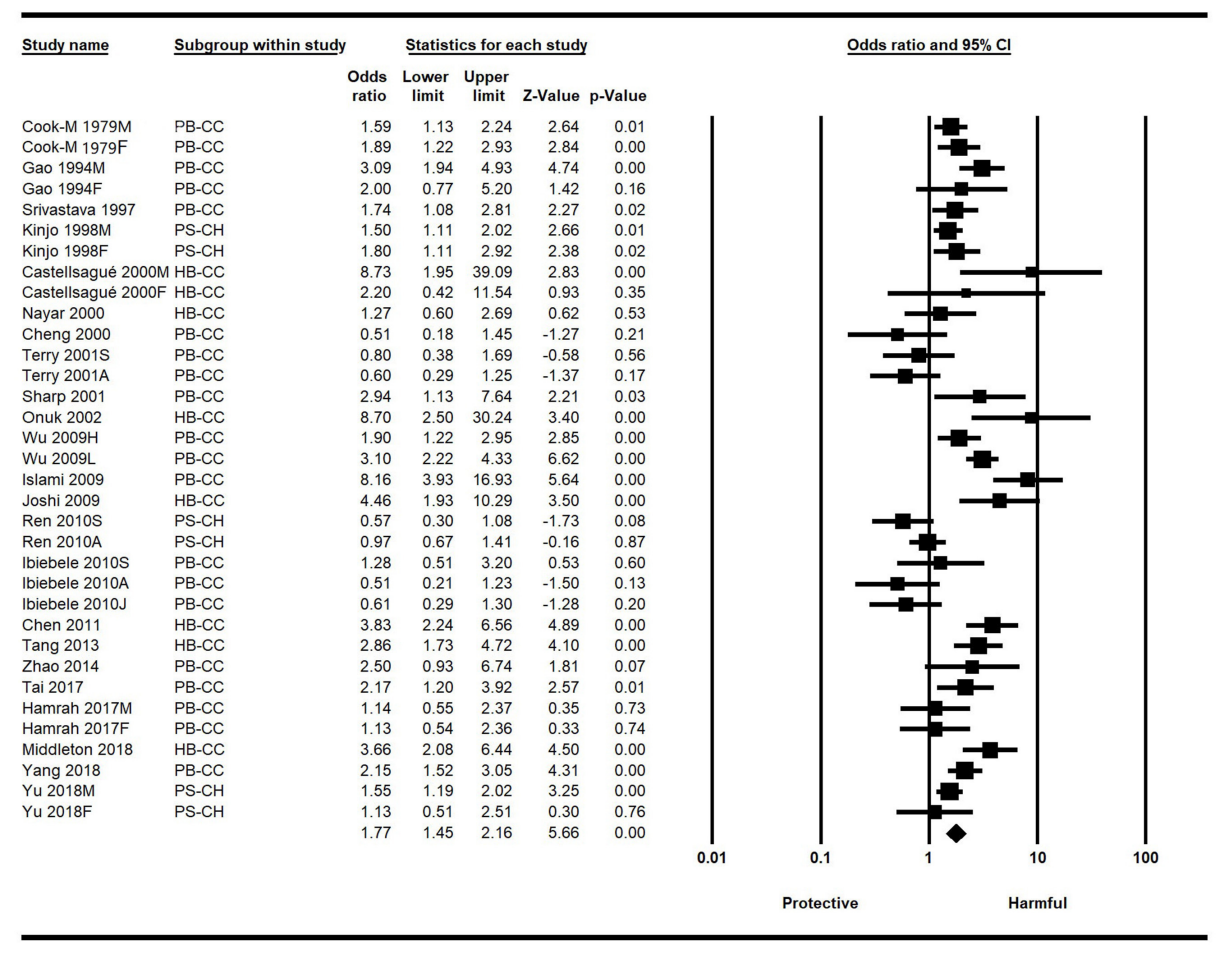
Forest plot of the effect of hot tea drinking on esophageal cancer (EC) risk based on the ORs and 95% CI. CI, confidence intervals; EC, esophageal cancer; HB-CC, hospital-based case control studies; ORs, odds ratios; PB-CC, population-based case control studies; PS-CH, prospective cohort studies.

Significant heterogeneity was observed among the included studies (I^2^ = 75.23%, *p* < 0.001), so the random-effects model was selected. The overall results of this meta-analysis showed that people who drink hot tea have a higher risk of EC than those who do not drink hot tea, with a combined OR value of 1.77 (95%CI: 1.45–2.16, *p* < 0.001), indicating that hot tea drinking can significantly increase the risk of EC (Figure 2).

### Subgroup Evaluation and Sensitivity Analysis

When we stratified the included studies according to the cancer types, the results of ESCC and EC were consistent within the overall articles. The pooled OR was 2.33 (95% CI: 1.51–3.61, *p* < 0.001) for ESCC, was 1.93 (95% CI: 1.61–2.32, *p* = 0.002) for combined EC. Five studies were included in the meta-analysis for EAC, comprising 680 cases and 1,313 controls. There was a statistically non-significant decreased risk of EAC in patients who consumed hot tea, with a pooled OR of.76 (95% CI = 0.58–1.01). There was no statistically heterogeneity (I^2^ = 0.00, *p* = 0.44) for EAC.

When we stratified the studies by different analyses design, the results of PB-CC studies (OR = 1.63, 95% CI: 1.26-2.12, *p* =0.001), HB-CC studies (OR = 3.36, 95% CI: 2.35–4.82, *p* = 0.11), and PS-CH studies (OR = 1.25, 95% CI: 0.95–1.65, *p* = 0.02) were all consistent within the overall conclusions (Table 2).

**Table 2:** Subgroup analyses according to potential sources of heterogeneity.

**Subgroups**	**Number**		**Meta–analyses**			**Heterogeneity**		**Model**
			**OR**	**95%CI**	**P**	**I^**2**^**	**P**	
Type of EC	ESCC	12	2.33	1.51–3.61	0.00	77.47	0.00	Random
	EAC	5	0.76	0.58–1.01	0.06	0.00	0.44	Fixed
	Combined	17	1.93	1.61–2.32	0.00	56.82	0.002	Random
Sex	Male	6	1.79	1.34–2.39	0.00	62.38	0.02	Random
	Female	6	1.66	1.28–2.16	0.00	0.00	0.76	Fixed
	Combined	24	1.78	1.31–2.41	0.00	81.84	0.00	Random
Study location	Asia	22	2.14	1.78–2.57	0.00	64.44	0.00	Random
	Europe	4	0.91	0.44–1.85	0.79	64.05	0.00	Random
	Africa	1	3.66	2.08–6.44	0.00	0.00	1.00	Fixed
	America	4	1.36	0.59–3.13	0.47	74.74	0.008	Random
	Australia	3	0.71	0.44–1.16	0.21	12.54	0.32	Fixed
Adjusted for confounders	Yes	25	1.80	1.41–2.31	0.00	78.41	0.00	Random
	No	9	1.71	1.23–2.38	0.00	63.79	0.005	Random
Control age	Yes	22	1.60	1.26–2.03	0.00	75.73	0.00	Random
	No	12	2.17	1.49–3.16	0.00	75.02	0.00	Random
Control smoking or alcohol	Yes	25	1.80	1.41–2.31	0.00	78.41	0.00	Random
	No	9	1.71	1.23–2.38	0.00	63.79	0.005	Random
Study design	PB-CC	20	1.63	1.26–2.12	0.00	74.03	0.00	Random
	HB-CC	8	3.36	2.35–4.82	0.00	39.80	0.11	Fixed
	PS-CH	6	1.25	0.95–1.65	0.02	61.98	0.02	Random

*EC, esophageal cancer; HB-CC, hospital-based case control studies; OR, odds ratio; PB-CC, population-based case control studies; PS-CH, prospective cohort studies*.

When we stratified the studies by different regions, the results of studies conducted in Asia (OR = 2.14, 95% CI = 1.78–2.57, *p* < 0.001), Africa (OR = 3.66, 95% CI = 2.08-6.44, *P* < 0.001), and the Americas (OR = 1.36, 95% CI = 0.59–3.13, *P* = 0.47) were consistent with the overall conclusion, while the results of those conducted in Europe (OR = 0.91, 95% CI = 0.44-1.85, *p* = 0.79) and Australia (OR =0 .71, 95% CI =0.44–1.16, *p* = 0.21) were the opposite (Table 2).

When stratified the studies by adjusting for confounders or not, the difference was also statistically significant between hot tea drinking and non/lowest level of hot tea consumption (Table 2).

To estimate the influence of each study on the overall effect size and to identify influential studies, we performed a leave-one-out meta-analysis by excluding one study at each analysis. For each study, the displayed effect size corresponds to an overall effect size computed from a meta-analysis excluding that study. The forest plot also displays a vertical line at the overall effect size based on the complete set of studies (with no omission) to help detect influential studies. Interestingly, when we omitted one study in turn, the ORs varied from 1.70 to 1.84, and the p-value was always < 0.001, which indicated that the overall result was robust (Supplementary Figure 1).

### Publication Bias

**Figure 3:**
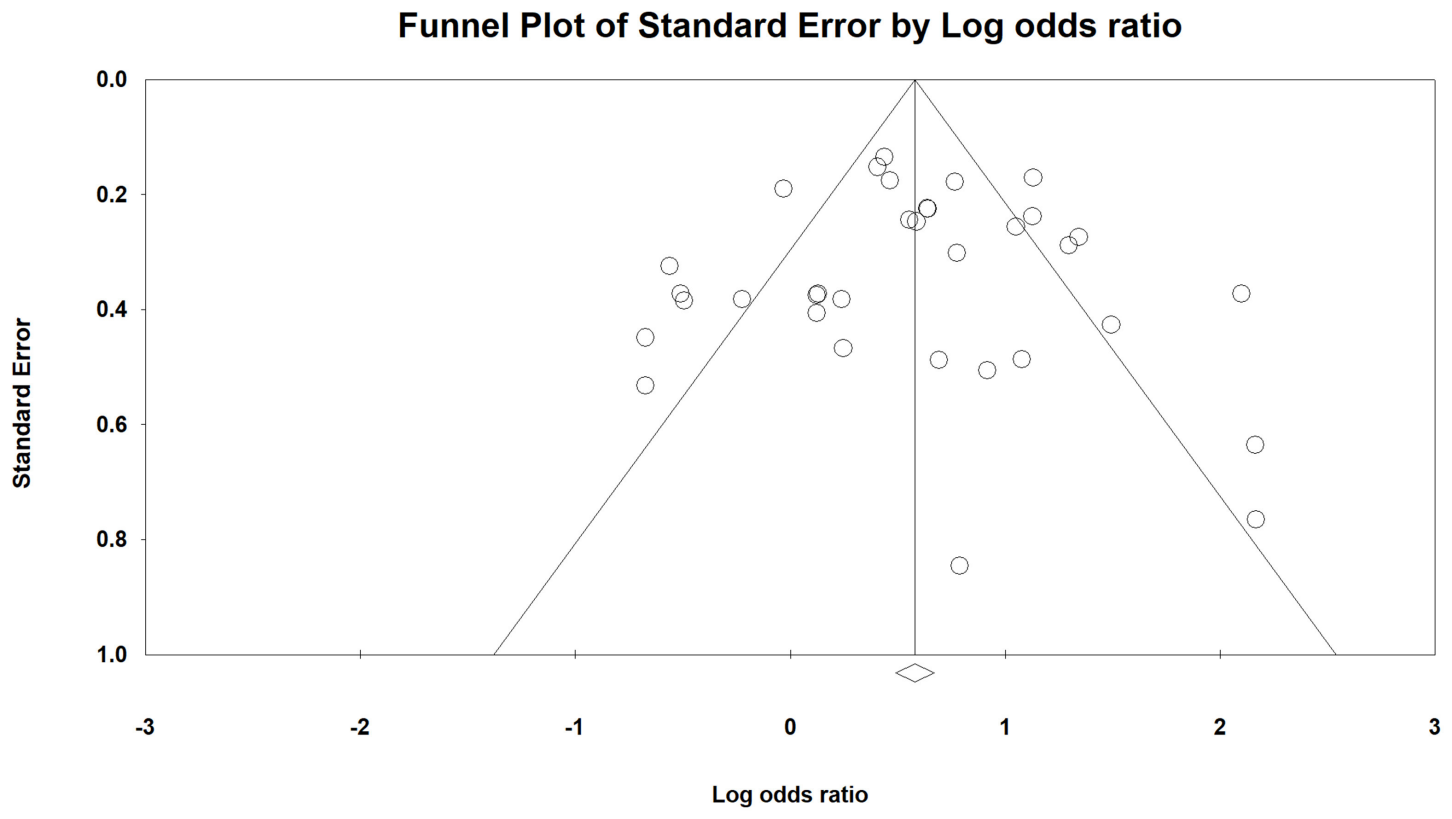
Funnel plot based on ORs of included studies. ORs, odds ratios.

Based on the visualization of the funnel plot (Figure 3), it was symmetrical, which indicated that there was no significant publication bias. The result was also confirmed by Egger's linear regression test (intercept = −0.003, t = 0.003, *p* = 1) and Begg's rank correlation test (Z = 0.00, *p* = 1).

## Discussion

This meta-analysis assessed the relationship between hot tea drinking and EC risk, according to 23 published case-control and cohort studies. The overall result indicated that drinking hot tea could significantly increase the risk of EC. A recent study also confirmed it was the temperature effect, but not polycyclic aromatic hydrocarbons (PAH) exposure that posed an EC risk (49). The increased risk seems to have been dominated by the ESCC subtype, which was significant even after adjusting for important confounders. This result is consistent with the majority of the literature to date (50, 51). However, studies are needed to explore why drinking hot tea increased the risk for ESCC but not for EAC.

Significant heterogeneity was observed in the overall meta-analysis for EC, and we failed to identify any individual study that was an important contributor to the heterogeneity by omitting one study in turn in the sensitivity analysis.

The subgroup analyses generated by stratifying the studies according to study design or sex were both consistent with the overall result. Publication bias was not present in both the ESCC and EAC subgroup analyses. However, the studies performed in Europe and Australia indicated a risk reduction trend, while in Asia, Africa, and America showed a significant trend of increased risk. The reason may be that EAC represents the majority of EC subtypes in these countries and this subtype is not easily influenced by hot temperature (4). Furthermore, the sample size for EAC is really small.

Notably, the association between hot tea drinking and EC risk may be affected by other confounding factors. For example, in Western populations, heavy tobacco smoking and alcohol consumption are the main risk factors for ESCC (52, 53). The EAC is strongly associated with Barrett's esophagus and gastroesophageal reflux disease (4). Therefore, a subgroup analysis of studies adjusting for smoking or alcohol consumption was conducted. The results remained similar, and the risk estimate was slightly increased (OR, 1.77 vs. 1.80), which indicated that smoking and alcohol drinking did not confound the results.

In previous studies, the tea temperature was mainly estimated by self-reported perception, the results may vary across individuals and could not be objectively verified. Besides, the existing evidence in humans for the carcinogenicity of drinking hot beverages is limited. According to the IARC classification system of carcinogens, “drinking very hot beverages at above 65°C” has been classified as “probably carcinogenic” (Group 2A), rather than “carcinogenic” to humans (Group 1) (54). Indeed, it was difficult to obtain the precise temperatures of hot tea in most of the studies. Tai et al. analyzed the risk of EC based on the tea temperature and found high temperature (≥60°C) significantly increases the risk of ESCC compared with low or mild temperature (<60°C) (47). Chen et al. reported a moderate risk EC when the tea temperature was 60-69°C, and the measured tea temperature above 70°C was associated with a high risk of EC (43). However, the methods used in these two studies were not precise enough and the measurement did not consider any changes in dietary habits or in temperature preferences in cancer cases that could happen due to the disease. Thus, further investigations are needed.

There were several limitations in this meta-analysis. Firstly, the heterogeneity could be eliminated, neither in overall results nor in most the subgroup analyses. For example, some studies only provided the unadjusted ORs, while others reported the adjusted ones. Moreover, the adjusted confounders were not always the same in different studies. All of these factors could explain the heterogeneity in the meta-analysis to some extent. Secondly, sufficient data were not provided in some literature. Lastly, most of the questionnaires used in the included studies were qualitative regarding the temperature of tea consumption, which was relied on self-reporting by the participants. Thus, we could not extract the exact temperature of tea drinking, and a more precise analysis could not be performed.

## Conclusions

This meta-analysis indicates that hot tea consumption is associated with a significantly increased risk in EC, particularly in ESCC. Given that hot tea consumption is prevalent in modern society, the results of our meta-analysis have important implications for EC etiology research as well as EC prevention.

## Data Availability Statement

The original contributions presented in the study are included in the article/Supplementary Material, further inquiries can be directed to the corresponding author.

## Author Contributions

HL designed the research, performed the literature search and statistical analysis, interpreted the data, and drafted the manuscript. HG designed the research, performed the literature search, handled funding, and made critical revisions of the manuscript. All authors read and approved the final manuscript.

## Funding

This work was supported by the National Natural Science Foundation of China (no. 81773230) and the Science and Technology Research Plan of Henan (no. 212102310619).

## Conflict of Interest

The authors declare that the research was conducted in the absence of any commercial or financial relationships that could be construed as a potential conflict of interest.

## Publisher's Note

All claims expressed in this article are solely those of the authors and do not necessarily represent those of their affiliated organizations, or those of the publisher, the editors and the reviewers. Any product that may be evaluated in this article, or claim that may be made by its manufacturer, is not guaranteed or endorsed by the publisher.

## Supplementary Material

Supplementary Figure 1 Forest plot of sensitivity analysis by omitting one study in turn. CI, confidence intervals; HB-CC, hospital-based case control studies; PB-CC, population-based case control studies; PS-CH, prospective cohort studies.
