# Supplementary figures and images for: Hot Tea Consumption and Esophageal Cancer Risk: A Meta-Analysis of Observational Studies
Source: Front Nutr. 2022 Apr 11;9:831567. doi: 10.3389/fnut.2022.831567 (PMC9035825; doi:10.3389/fnut.2022.831567)

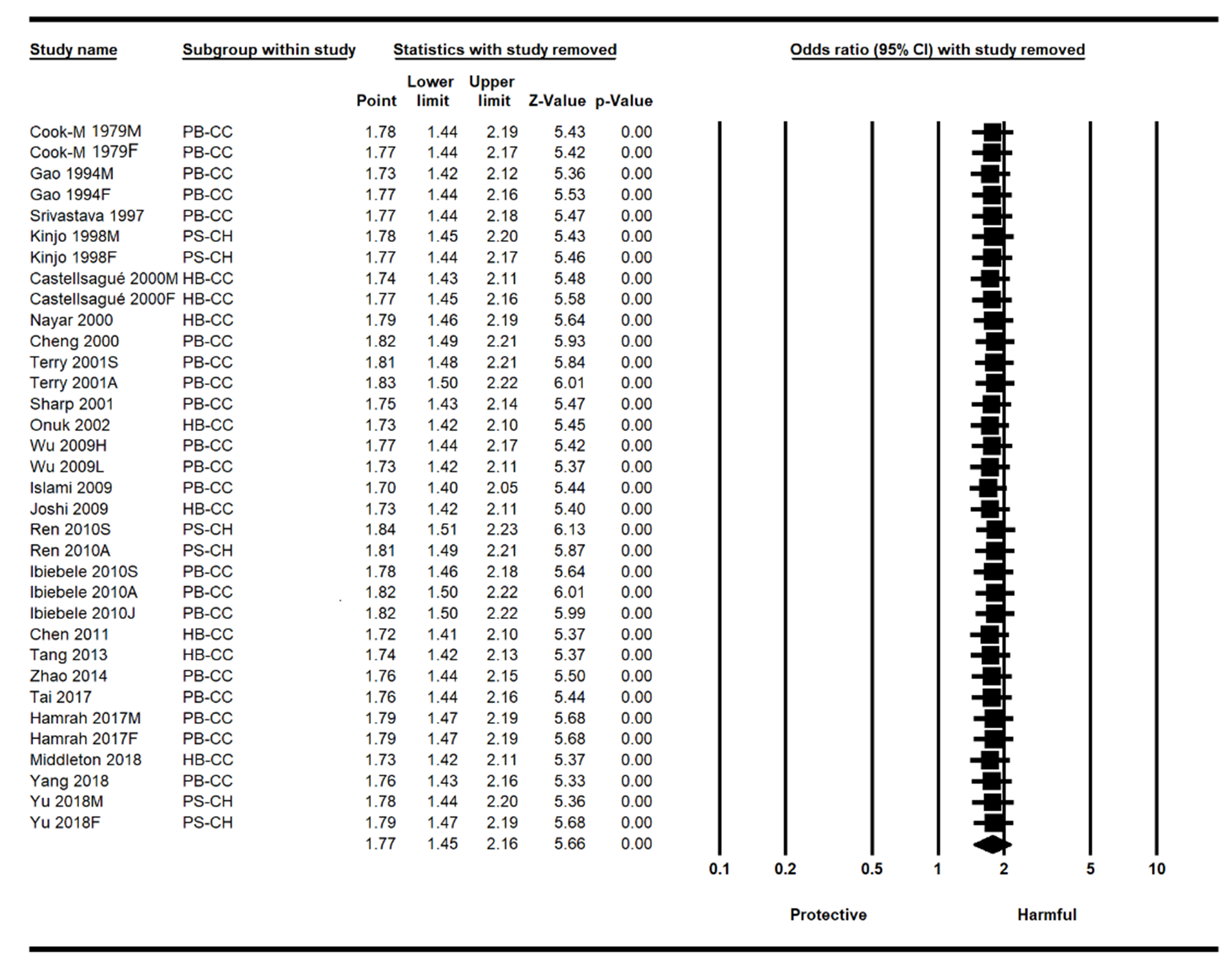

Supplement: Supplementary Figure 1 — Forest plot of sensitivity analysis by omitting one study in turn. CI, confidence intervals; HB-CC, hospital-based case control studies; PB-CC, population-based case control studies; PS-CH, prospective cohort studies. [file Image_1.TIF]
